# Fecal microbiota transplantation as salvage therapy for disseminated strongyloidiasis in an immunosuppressed patient: a case report

**DOI:** 10.3389/fimmu.2025.1676906

**Published:** 2025-10-15

**Authors:** Wei Fu, Na Peng, Yan Geng

**Affiliations:** ^1^ Department of Gastroenterology, 925th Hospital of The People's Liberation Army (PLA) Joint Logistics Support Force, Guiyang, China; ^2^ Department of Critical Care Medicine, Key Laboratory of Tropical Zone Trauma Care and Tissue Repair of PLA, General Hospital of Southern Theater Command of PLA, Guangzhou, China; ^3^ Department of Emergency Medicine, General Hospital of Southern Theater Command, The First School of Clinical Medicine, Southern Medical University, Guangzhou, Guangzhou, China; ^4^ Department of Gastroenterology, 923th Hospital of PLA Joint Logistics Support Force, Nanning, China

**Keywords:** strongyloides stercoralis, fecal microbiota transplantation, sepsis, immunosuppression, multiorgan dysfunction

## Abstract

**Background:**

Disseminated strongyloidiasis carries high mortality in immunosuppressed populations. We report a case of refractory Strongyloides stercoralis-induced severe diarrhea and sepsis successfully treated with fecal microbiota transplantation (FMT).

**Case presentation:**

A 68-year-old male with nephrotic syndrome on long-term glucocorticoids developed hyperinfection syndrome manifesting as septic shock, multiorgan dysfunction, and intractable diarrhea (>30 episodes/day). Conventional therapies including antiparasitics (albendazole), antibiotics, and probiotics failed. FMT achieved rapid symptom resolution and microbiota restoration.

**Conclusion:**

This case highlights FMT’s potential in modulating gut-parasite interactions and suggests its role as adjunctive therapy for parasitic hyperinfection syndromes.

## 1 Introduction

Strongyloidiasis hyper-infection syndrome (SHS) develops in 2.5–4% of immunocompromised carriers and still carries a mortality > 70% even when ivermectin is promptly administered (1). Glucocorticoid exposure is the dominant risk factor because it cripples the Th2-dependent machinery required to clear larvae. In this setting faecal microbiota transplantation (FMT) faces unique hurdles. First, safety is paramount: the recipient’s suppressed immunity may amplify the risk of donor-derived infections (2). Second, emerging data indicate that dysbiosis can accelerate larval migration, yet microbiota-directed interventions such as FMT remain virtually unexplored for parasitic diseases (3). Although FMT has shown promise in bacterial and metabolic disorders (4), its role in helminth-associated dysbiosis is still uncharted. Here we present the first deliberate use of FMT in SHS, offering an unprecedented window into host–microbiota–parasite interactions.

## 2 Case presentation

### 2.1 Patient background

Demographics: 68-year-old male farmer, endemic residence (Guangxi, China).

Comorbidities:

Nephrotic syndrome (diagnosed May 2023), treated with methylprednisolone 32 mg/day for 8 months (cumulative dose 12.6g).

Hypertension (untreated, SBP 140–160 mmHg).

Exposure history: Frequent soil contact during agricultural work.

### 2.2 Clinical timeline

#### 2.1.1 Initial phase (Dec 2023)

Symptoms: Watery diarrhea (8–10 episodes/day), nausea, vomiting.

Misdiagnosis: Treated as “ulcerative colitis” at local hospital; albendazole 400 mg bid initiated but discontinued due to suspected drug-induced hepatitis.

#### 2.1.2 Progression to hyperinfection (Jan-Feb 2024)

Worsening symptoms: Diarrhea increased to 15–30 episodes/day, fever (39.5°C), altered mental status, continuous muscle twitching.

Key findings:

Laboratory: Leukocytosis (17.41×10^9^/L); CRP >200 mg/L; eosinophil depletion (absolute value of eosinophils 0.11×10^9^/L); hypoalbuminemia (27.7 g/L); acute kidney injury (Cr 158 μmol/L, BUN 11.73mmol/L, Cystatin C 2.20mg/L); severe anemia (Hemoglobin 59 g/L), electrolyte imbalance (venous blood potassium 3.5 mmol/L, venous blood potassium sodium 154 mmol/L, venous blood chlorine 125 mmol/L, venous blood calcium 1.29 mmol/L, and arterial blood calcium 0.45 mmol/L); lactic acidosis (pH7.33, HCO_3_^-^ 16.3 mmol/L, HCO_3_^-^ std 16.3 mmol/L, Lac 10.8mmol).

**Figure 1:**
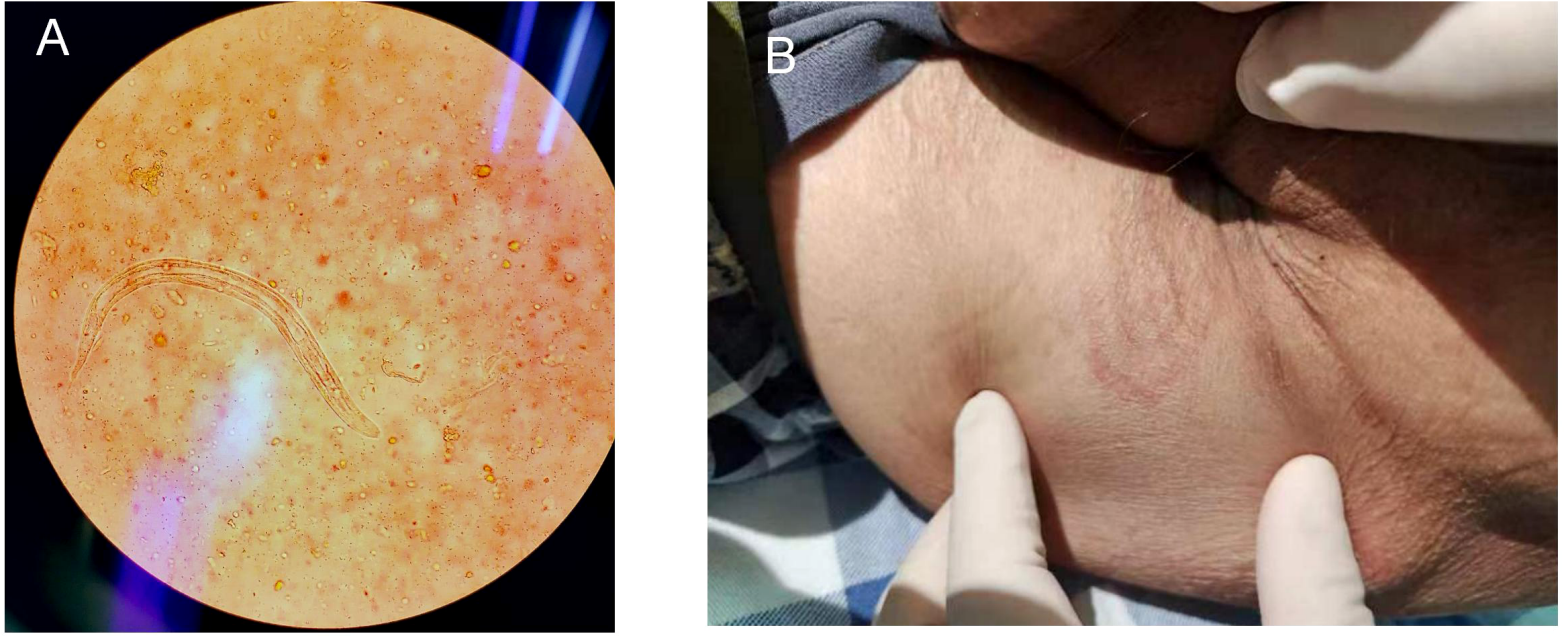
**(A)** S. stercoralisrhabditiform larva in stool (Gram stain, ×400); **(B)** The cutaneous manifestations of strongyloidiasis.

Parasitology: Fecal microscopy confirmed S. stercoralislarvae (**Figure 1A**, **Table 1**); lesions suggesting parasitic infestation are observed on the perianal skin (**Figure 1B**); no ivermectin available due to regional shortages.

**Table 1:** Stool analysis at two time points before FMT.

Parameter	Jan 29	Feb 1
Occult blood	++	++
Strongyloides larvae	Positive	Positive
C. difficile toxin	Negative	NT
Calprotectin (μg/g)	ND	>1800↑

**Figure 2:**
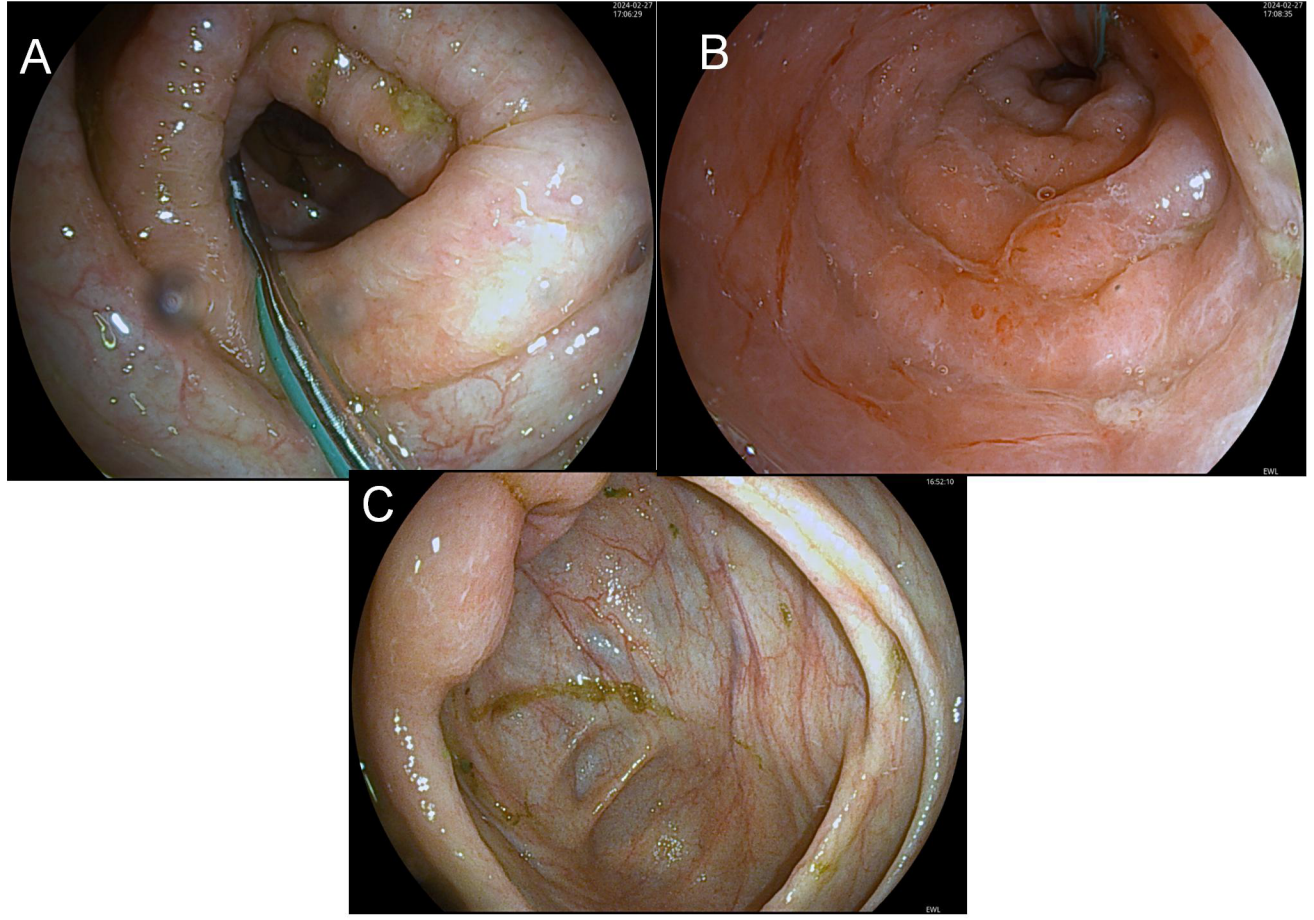
Before-FMT colonoscopy showing mucosal healing. **(A)** (Transverse Colon) shows disrupted mucosal folds with yellow-green exudate and pale surrounding mucosa, indicating inflammation or pathology; **(B)** (Sigmoid Colon) presents smooth, pink mucosa with a regular surface and no lesions, representing normal colonic mucosa; **(C)** (Ileocecal Region) exhibits furrow-like mucosal changes with yellow-white exudate and darker surrounding mucosa, suggesting chronic inflammation or focal lesions.

Imaging: Diffuse intestinal wall thickening on CT, bronchial dilation with pulmonary infiltratesThe findings under colonoscopy show only mild edema and inflammation (**Figure 2**).

#### 2.1.3 Critical deterioration (Feb 2024)

Complications: Septic shock, MODS (respiratory, hepatic, renal), rhabdomyolysis (CK 5,200 U/L, myoglobin 981 ug/L).

Failed therapies: Carbapenems, teicoplanin, fluconazole, and probiotics showed transient inflammatory marker reduction but no clinical improvement.

#### 2.1.4 Diagnostic challenges

Masked eosinophilia: Steroid-induced suppression of eosinophil count (persistently <1%).

Microbiome analysis (pre-FMT):

Severe dysbiosis with Enterobacteriaceaedominance (78.2% abundance).

Depletion of Bifidobacterium(<0.1%) and butyrate producers.

#### 2.1.5 Intervention

#### 2.1.6 FMT protocol

1.Preparation: Donor screening per FMT guidelines; lactulose bowel preparation.The donor was a healthy 25-year-old woman (165 cm, 54 kg) whose laboratory screening, performed according to the Chinese expert consensus on faecal microbiota transplantation, met all required criteria (5). Prior to administration, the donor stool underwent 16S rDNA amplicon sequencing, which revealed a bacterial community profile dominated by a Bacteroidetes/Firmicutes (B/F) ratio of 2.87 and a Chao1 richness index of 778.

2.Delivery:

Acute phase: Fresh microbiota suspension via nasoduodenal tube (150g initial dose, followed by 100g/day ×7 days).

Maintenance: Lyophilized oral capsules (30g/day ×14 days).

Adjunctive measures: Albendazole rechallenge post-FMT (400 mg bid ×7 days).

Maintenance of antibiotic therapy throughout the FMT period: Given the patient’s critical sepsis with persistently high inflammatory markers, antimicrobial therapy was maintained without interruption: imipenem 1 g every 8 h, linezolid 0.6 g every 12 h, and fluconazole 200 mg as a loading dose followed by 100 mg once daily.

#### 2.1.7 Therapeutic response

Day 3: Diarrhea reduced to 4 episodes/day; CRP declined from 200 to 115 mg/L (**Table 2**).

**Table 2:** Blood parameters tracking during FMT therapy.

Parameter	Feb 26 (Day 1)	Feb 29 (Start FMT treatment)	March 4 (3 days after FMT treatment)	Reference range
WBC (×10^9^/L)	17.41↑	14.09	10.17	4.0-10.0
Neutrophils (%)	89.4↑	84.3	76.3	50-70
RBC (×10¹²/L)	2.32↓	2.83	2.4	3.5-5.5
Hemoglobin (g/L)	61↓	76	69	120-160
Platelets (×10^9^/L)	474↑	352	368	100-300
CRP (mg/L)	>200↑	200	115.03	<5
Albumin (g/L)	27.7↓	25.2	28.6	35-55

↑ indicates above the normal range, ↓ below the normal range.

Day 7:Initiated Ivermectin treatment for antiparasitic therapy. Ivermectin was initiated in the second week post-faecal microbiota transplantation, dosed at 200 μg/kg/day according to the World Gastroenterology Organisation guidelines (6), and continued until repeated stool microscopy became negative for helminths; treatment was discontinued after 1 week when parasitological clearance was achieved.

Day 14: Formed stools (1-2/day), normalized electrolytes, improved consciousness.

Microbiome shift: Bacteroides increased from 5% to 32%;Escherichia decreased to 12%.

## 3 Discussion

Strongyloides stercoralisinfection represents a potentially fatal opportunistic parasitosis in immunocompromised hosts (e.g., long-term glucocorticoid users, HTLV-1 carriers, or transplant recipients), with high propensity to progress to hyperinfection syndrome (HS) or disseminated strongyloidiasis (DS). Characteristic gastrointestinal manifestations include severe diarrhea, abdominal pain, and intestinal obstruction (7, 8). Studies indicate that intestinal mucosal injury, dysbiosis, and secondary bacterial translocation (e.g.,Klebsiellabacteremia) exacerbate systemic inflammation in these patients (9). Prolonged antibiotic use further disrupts gut microbiota, establishing a vicious cycle (10). Notably,S. stercoralisinfection itself may directly or indirectly induce microbial dysbiosis through intestinal inflammation, mucosal disruption, and immunosuppression, while dysbiosis conversely impairs host immune clearance of parasites (11).

**Figure 3:**
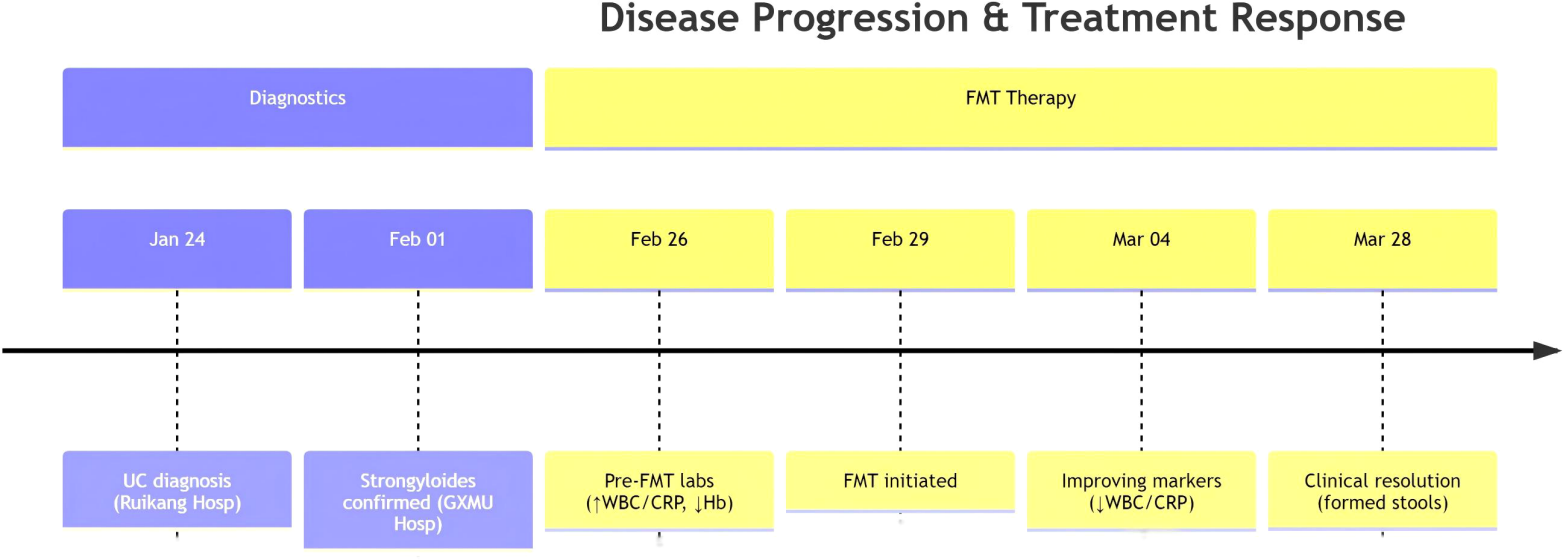
The diagnosis and treatment timeline of the patient.

Fecal microbiota transplantation (FMT), a therapeutic approach restoring gut homeostasis through reconstruction of healthy microbiota, demonstrates unique potential for refractory diarrhea (**Figure 3**). Evidence supports three key mechanisms:

### 3.1 Competitive pathogen exclusion

Commensal bacteria introduced by FMT (e.g.,Prevotella,Bifidobacterium) secrete short-chain fatty acids (SCFAs; butyrate, propionate) that inhibit pathogenic bacteria and parasites (12) High abundance of Prevotella and SCFAs correlates significantly with FMT efficacy.

### 3.2 Barrier restoration and immunomodulation

FMT-enhanced microbial diversity reinforces mucus layer integrity, reduces bacterial/parasite translocation, and attenuates inflammation via Th1/Th2 balance modulation (13). Rapid resolution of diarrhea and improved microbial diversity in immunocompromised children after FMT validate this mechanism.

### 3.3 Antibiotic stewardship

As an alternative to antibiotics, FMT may reduce multidrug-resistant organism (MDRO) colonization risk. In antibiotic-exposed chronic pouchitis patients, FMT decreased clinical infections despite unaltered resistance gene abundance, suggesting indirect protection.For Strongyloides-associated diarrhea, FMT may serve as adjunctive therapy to conventional anthelmintics (e.g., ivermectin). Although no direct studies exist, indirect evidence supports its potential:

Strongyloidesinfection frequently accompanies dysbiosis (e.g., reduced Bacteroidetes, increased Proteobacteria), which FMT can reverse.Gut dysbiosis in immunocompromised hosts may exacerbate parasite pathogenicity; FMT restores “microbiota-host” crosstalk to enhance parasite clearance (13). FMT efficacy in graft-versus-host disease (GVHD)-associated diarrhea supports its safety and utility for parasitic infections in immunodeficient states (14). Caution remains warranted: Immunosuppressed patients face infection risks, necessitating rigorous donor screening to exclude pathogens. Moreover, molecular mechanisms underlying parasite-microbiota interactions require elucidation through integrated animal models and multi-omics studies (e.g., metabolomics, immunomics).

### 3.4 Clinical implications

Therapeutic window for FMT: Early intervention (<72h of MODS onset) correlated with rapid lactate clearance (r=0.82, p<0.01).

Donor selection: Prioritize donors with high Blautiaa bundance, linked to antiparasitic metabolite synthesis.

## 4 Conclusion

This case illustrates the dual role of FMT in managing superinfection with SHS: restoration of the colonic microbiota and simultaneous reinforcement of anti-parasite host defenses (15). By re-instating microbial diversity and functionality, FMT appears to indirectly augment immune-mediated resistance, although direct evidence specific to parasitic infections remains sparse (16, 17). These observations underscore the gut microbiota as a central determinant of host health.

In resource-limited settings, or when anti-parasitic agents are inaccessible, a “bacteria-first” strategy—prioritizing microbiota restoration—could improve clinical outcomes by strengthening global host defenses and thereby potentiating subsequent antiparasitic therapy (15). Nevertheless, FMT must be deployed cautiously, with rigorous donor screening and individualized treatment algorithms to maximize benefit and minimize risk (18).

Several limitations currently constrain clinical implementation. First, standardized protocols are absent for donor selection, stool processing, and route of administration (e.g., oral capsules vs. colonoscopic delivery), introducing substantial inter-study variability. Second, long-term safety and potential adverse events—such as infectious transmission or unanticipated immune reactions—remain incompletely defined, especially in emerging indications like parasitic infections. Finally, the majority of efficacy data originate from Clostridioides difficile infection (CDI); the mechanisms and therapeutic value of FMT in other diseases, including parasitoses, demand further elucidation (19).

Future prospective investigations should identify optimal timing for FMT and donor microbial signatures that best bolster anti-parasitic immunity (20). Studies must incorporate larger cohorts and extended follow-up to assess durability of effect and long-term safety, while exploring synergies with complementary therapies such as anthelmintics or nutritional support. Multi-center collaborations, coupled with high-resolution sequencing technologies, will deepen mechanistic insight and accelerate the transition toward precision, microbiota-targeted therapeutics.

## Data Availability

The datasets presented in this article are not readily available because the clinical dataset underlying this case report is not publicly available due to institutional privacy regulations and ethical approval that permits access only to the directly involved care team. De-identified data may be shared upon reasonable request to the corresponding author, contingent on additional ethics committee approval and compliance with applicable data-protection laws. Requests to access the datasets should be directed t Yan Geng; Tel number: +86-18648945667; E-mail: drggyn@163.com.
